# An Infant With Hyper IgE Syndrome Mimicking Acute Leukemia

**DOI:** 10.1002/ccr3.72409

**Published:** 2026-04-01

**Authors:** Yuichiro Matsui, Suguru Uemura, Kenji Kishimoto, Kana Hamada, Naoya Morisada, Daiichiro Hasegawa, Yoshiyuki Kosaka

**Affiliations:** ^1^ Department of Hematology and Oncology Kobe Children's Hospital Kobe Japan; ^2^ Department of Allergology Kobe Children's Hospital Kobe Japan; ^3^ Department of Clinical Genetics Kobe Children's Hospital Kobe Japan

**Keywords:** eosinophilia, hyper IgE syndrome, leukemoid reaction, STAT3

## Abstract

Although infection, inflammation, and leukemia are major causes of leukocytosis, hyper IgE syndrome should be considered if eosinophilia is present. Hyper IgE syndrome may mimic acute leukemia when complicated by severe infection.

## 1 Introduction

Hyper IgE syndrome (HIES) comprises a group of rare primary immunodeficiency disorders characterized by a triad of atopic dermatitis, recurrent eczema, and pulmonary infections along with elevated IgE levels [1]. Autosomal dominant and loss‐of‐function pathogenic variants in the *STAT3* gene located on chromosome 17 are the most common causes of this disease [2]. Eosinophilia is noted in 80% of HIES patients with *STAT3* mutations [3]. In the patients with HIES, eosinophilia usually occurs due to a Th2‐dominant deviation caused by loss of STAT3 function [4]. A scoring system developed by the National Institutes of Health (NIH) is valid [5]; a score of 40 or higher is sufficient to diagnose HIES, although it is often difficult to diagnose in infancy.

A leukemoid reaction is a reactive leukocytosis which can mimic leukemia due to an underlying condition [6] and is characterized by more than 5.0 × 10^9^/L of a white blood cell (WBC) count with a shift to the left. This response is considered benign because the dramatic changes in WBC count resolve with improvement in the underlying etiology. Previous studies of leukemoid reaction in childhood have described that the most common etiology identified was infectious disease, which accounted for about 50% of cases [7, 8].

There are few reports of HIES complicated by leukemoid reaction. HIES can be complicated by severe infections that result in marked leukocytosis, while WBC count in patients with HIES is usually normal, but there can be relative neutropenia [9]. Here, we describe an infant case who required a differential diagnosis of leukemia and was later diagnosed with HIES.

## 2 Clinical Case Presentation

**FIGURE 1:**
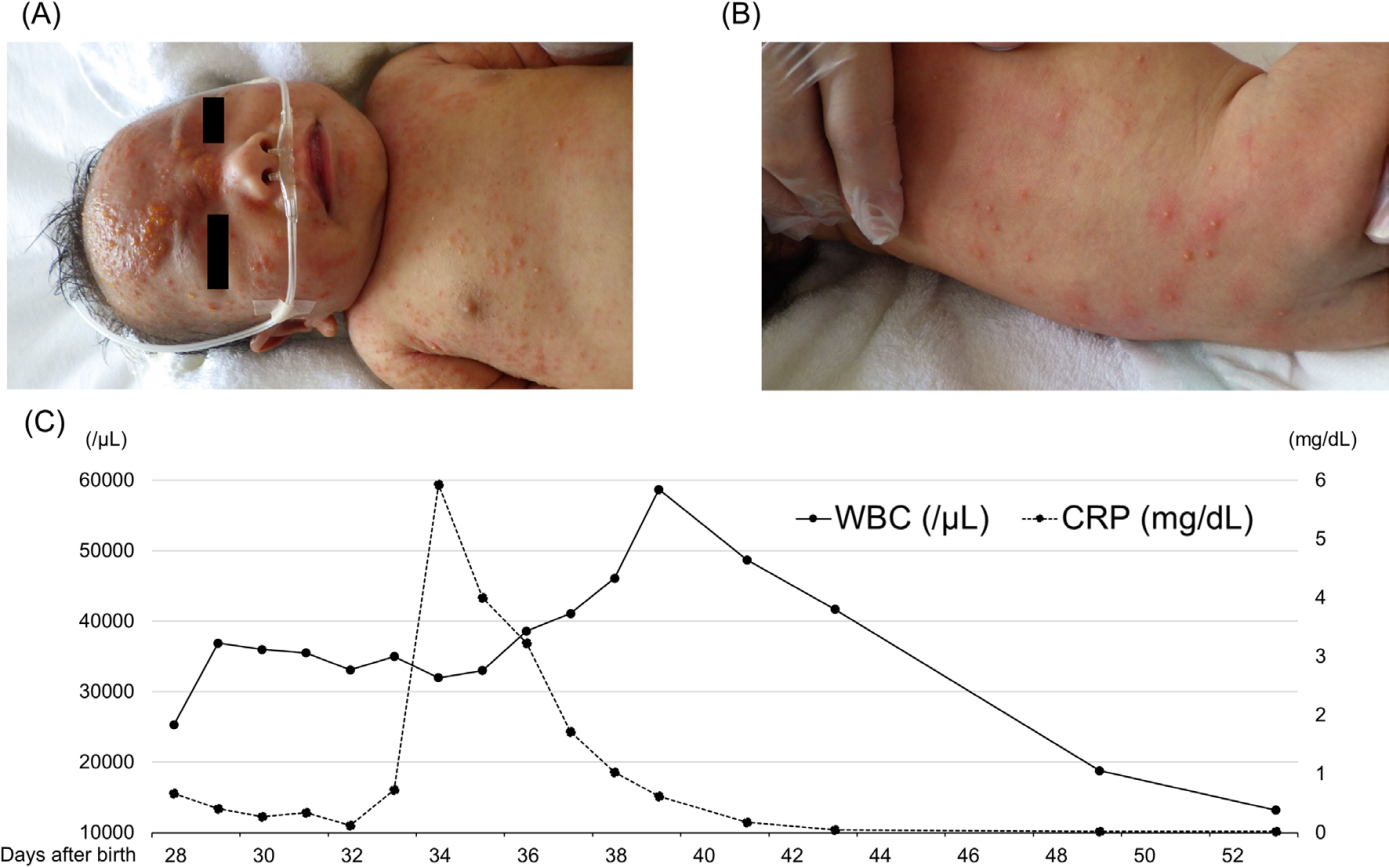
Clinical findings of the present case. (A, B) Crusted papulopustular eruption at admission. (A) Face to trunk and (B) Back. (C) Clinical course of the present case. C‐reactive protein, CRP; white blood cell, WBC.

A 38‐day‐old male infant was referred to our hospital for treatment of generalized pustules with effusion and hyperleukocytosis. His family history was unremarkable. His mother was aged 29 years (gravid 2, para 2) and her pregnancy was uncomplicated. He was born at term by normal vaginal delivery. His birth weight was 2894 g. Although he was fed formula for allergies because of facial eczema from day 14, the eczema became widespread. He was admitted to the referral hospital on day 28, presenting with numerous pustules with effusion, particularly on the face, as well as edema and fever (Figure 1A,B). Laboratory tests revealed a WBC count of 25.3 × 10^9^/L (eosinophils, 38.0%), hemoglobin level of 13.1 g/dL, and platelet count of 128 × 10^9^/L. Serum lactose dehydrogenase (LDH) was 446 U/L. C‐reactive protein (CRP) was 0.67 mg/dL. Due to the suspicion of staphylococcal scalded skin syndrome, vancomycin was initiated. Methicillin‐resistant *Staphylococcus aureus* was isolated from an exudate sample collected from a pustular lesion. No pathogens were detected in blood cultures collected at admission. After admission, the WBC count, LDH, and CRP levels gradually increased, and the hemoglobin level and platelet count decreased (Figure 1C). A red blood cell transfusion was performed on day 34 because the hemoglobin level decreased to 6.8 g/dL.

On admission to our hospital on day 38, the laboratory tests revealed a WBC count of 38.6 × 10^9^/L, a hemoglobin level of 11.1 g/dL, and a platelet count of 45 × 10^9^/L. A differential WBC count on a peripheral blood smear showed 0.5% myeloblasts, 0.5% promyelocytes, 2.0% myelocytes, 0.5% metamyelocytes, 6.0% band forms, 26.5% neutrophils, 39.0% eosinophils, and 25.0% lymphocytes. The IgE level was 44 IU/mL, which was above the age‐appropriated reference range [10]. A bone marrow examination was performed on day 41 to exclude acute leukemia, because marked leukocytosis exceeding 50 × 10^9^/L was accompanied by anemia and thrombocytopenia, despite the absence of an increased blast population in the peripheral blood. The bone marrow examination revealed a nuclear cell count of 65 × 10^9^/L without an increase in blasts or evidence of cellular dysplasia. Cytogenetic analysis using G‐banding showed a normal karyotype. Vancomycin and skin care, including topical steroids, resulted in the gradual improvement of his eczema. Additionally, the WBC count became normalized, and the hemoglobin level and platelet count increased. Although he was discharged from our hospital on day 56, the IgE levels gradually increased, and eosinophilia persisted. The eczema also repeatedly recurred. Because hyper IgE syndrome (HIES) was suspected based on high level of IgE and repeated eczema, targeted gene sequencing of *STAT3, TYK2, IL6R, ZNF341, ERBIN, TGFBR1, TGFBR2, SPINK5, PGM3, CARD11*, and *DOCK8* was performed on his peripheral blood sample, revealing the presence of a heterozygous mutation, c.1909G>A (p.Val637Met) in *STAT3* (NM_139276.3). This variant in *STAT3* was classified as pathogenic in the database of ClinVar (https://www.ncbi.nlm.nih.gov/clinvar/) and previously reported [11]. He was diagnosed with HIES at the age of 12 months. Retrospectively, the NIH clinical HIES scoring was calculated [5]. The score on admission and at diagnosis with HIES were 25 points and 37 points, respectively. The IgE level at the diagnosis with HIES was 4990 IU/mL.

## 3 Discussion

Here, we describe an infantile case of HIES mimicking acute leukemia. In the present case, hyperleukocytosis was thought to be due to a leukemoid reaction. In this case, the WBC count continued to increase despite a decline in CRP levels. In patients with STAT3‐deficient HIES, impaired Th17 differentiation and defective IL‐17–mediated neutrophil recruitment result in dysregulated inflammatory responses [12]. Although neutrophil production in the bone marrow is preserved, neutrophil migration to sites of infection is impaired, which may lead to persistent or progressive leukocytosis even as systemic inflammatory markers such as CRP begin to decrease. In addition, a Th2‐skewed immune environment with marked eosinophilia may have contributed to the sustained elevation of the total WBC count in the present case. The associated anemia and thrombocytopenia were considered secondary to severe infection. Consistent with previous experimental findings showing preserved granulopoiesis in STAT3‐deficient mice, the marked neutrophilia with a left shift observed in this case may have been driven by an unresolved severe bacterial infection [13].

In the previous pediatric report with hyperleukocytosis (defined as WBC count ≥ 30 × 10^9^/L), the patients with a WBC count ≥ 50 × 10^9^/L had a sixfold higher risk of leukemia than those with a WBC count < 50 × 10^9^/L [14]. In the present case, bone marrow examination did not reveal findings consistent with leukemia, and his complete blood count parameters gradually normalized. Thus, the possibility of leukemia was ruled out. A large cohort with HIES in Iran showed that the mean WBC at diagnosis was 11.5 × 10^9^/L (interquartile range: 8.8–15.8 × 10^9^/L) [15]. On the other hand, Chamlin SL, et al. reported three of eight patients with HIES had leukocytosis (WBC count: > 24 × 10^9^/L) prior to diagnosis [7]. Although infection, inflammation, and leukemia are major causes of leukocytosis, HIES should be considered if eosinophilia is present. It should be noted that serum IgE levels in patients with HIES, particularly during early infancy, may be within or only slightly above the age‐adjusted reference range at initial presentation and may increase progressively over time. Therefore, reliance on serum IgE levels alone may delay the diagnosis of HIES in infants. In such cases, clinical features such as early‐onset eczema and persistent eosinophilia should prompt consideration of HIES even when IgE levels are not markedly elevated [4].

In conclusion, HIES may mimic acute leukemia when complicated by severe infection. Bone marrow examination should be performed to confirm the diagnosis in such cases.

## Author Contributions

**Yuichiro Matsui:** conceptualization, data curation, writing – original draft. **Suguru Uemura:** conceptualization, data curation, supervision, writing – original draft. **Kenji Kishimoto:** writing – review and editing. **Kana Hamada:** writing – review and editing. **Naoya Morisada:** writing – review and editing. **Daiichiro Hasegawa:** writing – review and editing. **Yoshiyuki Kosaka:** supervision, writing – review and editing.

## Funding

The authors have nothing to report.

## Consent

Written informed consent for publication of the case and images was obtained from the patient prior to the writing of this case report.

## Conflicts of Interest

The authors declare no conflicts of interest.

## Data Availability

The data that support the findings of this study are availableon request from the corresponding author. The data are notpublicly available due to privacy or ethical restrictions.

## References

[1] B. Grimbacher , S. M. Holland , J. I. Gallin , et al., “Hyper‐IgE Syndrome With Recurrent Infections — An Autosomal Dominant Multisystem Disorder,” New England Journal of Medicine 340, no. 9 (1999): 692–702. 10053178 10.1056/NEJM199903043400904

[2] Y. Minegishi , M. Saito , S. Tsuchiya , et al., “Dominant‐Negative Mutations in the DNA‐Binding Domain of STAT3 Cause Hyper‐IgE Syndrome,” Nature 448, no. 7157 (2007): 1058–1062. 17676033 10.1038/nature06096

[3] M. O. Chandesris , I. Melki , A. Natividad , et al., “Autosomal Dominant STAT3 Deficiency and Hyper‐IgE Syndrome: Molecular, Cellular, and Clinical Features From a French National Survey,” Medicine (Baltimore) 91, no. 4 (2012): e1–e19. 22751495 10.1097/MD.0b013e31825f95b9 PMC3680355

[4] Y. Minegishi , “Hyper‐IgE Syndrome, 2021 Update,” Allergology International 70, no. 4 (2021): 407–414. 34419355 10.1016/j.alit.2021.07.007

[5] B. Grimbacher , A. A. Schäffer , S. M. Holland , et al., “Genetic Linkage of Hyper‐IgE Syndrome to Chromosome 4,” American Journal of Human Genetics 65, no. 3 (1999): 735–744. 10441580 10.1086/302547 PMC1377980

[6] J. P. Portich and G. A. M. Faulhaber , “Leukemoid Reaction: A 21st‐Century Cohort Study,” International Journal of Laboratory Hematology 42, no. 2 (2020): 134–139. 31765058 10.1111/ijlh.13127

[7] M. Brauner , M. Goldman , and E. Kozer , “Extreme Leucocytosis and the Risk of Serious Bacterial Infections in Febrile Children,” Archives of Disease in Childhood 95, no. 3 (2010): 209–212. 20308337 10.1136/adc.2009.170969

[8] V. Sakka , S. Tsiodras , E. J. Giamarellos‐Bourboulis , and H. Giamarellou , “An Update on the Etiology and Diagnostic Evaluation of a Leukemoid Reaction,” European Journal of Internal Medicine 17, no. 6 (2006): 394–398. 16962944 10.1016/j.ejim.2006.04.004

[9] A. F. Freeman and S. M. Holland , “Clinical Manifestations, Etiology, and Pathogenesis of the Hyper‐IgE Syndromes,” Pediatric Research 65, no. 5 Pt 2 (2009): 32R–37R. 10.1203/PDR.0b013e31819dc8c5 PMC2919366 19190525

[10] T. B. Martins , M. E. Bandhauer , A. M. Bunker , W. L. Roberts , and H. R. Hill , “New Childhood and Adult Reference Intervals for Total IgE,” Journal of Allergy and Clinical Immunology 133, no. 2 (2014): 589–591. 24139495 10.1016/j.jaci.2013.08.037

[11] S. M. Holland , F. R. DeLeo , H. Z. Elloumi , et al., “STAT3 Mutations in the Hyper‐IgE Syndrome,” New England Journal of Medicine 357, no. 16 (2007): 1608–1619. 17881745 10.1056/NEJMoa073687

[12] Y. Minegishi , M. Saito , M. Nagasawa , et al., “Molecular Explanation for the Contradiction Between Systemic Th17 Defect and Localized Bacterial Infection in Hyper‐IgE Syndrome,” Journal of Experimental Medicine 206, no. 6 (2009): 1291–1301. 19487419 10.1084/jem.20082767 PMC2715068

[13] A. D. Panopoulos , L. Zhang , J. W. Snow , et al., “STAT3 Governs Distinct Pathways in Emergency Granulopoiesis and Mature Neutrophils,” Blood 108, no. 12 (2006): 3682–3690. 16888100 10.1182/blood-2006-02-003012 PMC1895456

[14] A. Hoofien , H. Yarden‐Bilavski , S. Ashkenazi , G. Chodick , and G. Livni , “Leukemoid Reaction in the Pediatric Population: Etiologies, Outcome, and Implications,” European Journal of Pediatrics 177, no. 7 (2018): 1029–1036. 29696475 10.1007/s00431-018-3155-5

[15] M. Tavassoli , H. Abolhassani , R. Yazdani , et al., “The First Cohort of Iranian Patients With Hyper Immunoglobulin E Syndrome: A Long‐Term Follow‐Up and Genetic Analysis,” Pediatric Allergy and Immunology 30, no. 4 (2019): 469–478. 30801830 10.1111/pai.13043

